# Meta-analysis of mean differences from randomized trials with nested clustering

**DOI:** 10.1186/1745-6215-14-S1-P125

**Published:** 2013-11-29

**Authors:** Rebecca Walwyn

**Affiliations:** 1 University of Leeds, Leeds, UK

## 

Nesting of patients within care providers in trials of physical and talking therapies creates an additional level within the design. The statistical implications of this are analogous to those of cluster-randomised trials, except that the clustering effect interacts with treatment, leading to different ICCs across treatment arms. In some cases, the clustering effect may be restricted to one or more of the arms, resulting in a "partially nested design". The statistical model that is recommended at the trial-level includes a random effect for the care provider but allows the provider and patient level variances to differ across arms. The care provider variance is constrained to equal zero where there is no clustering in partially nested trials. Evidence suggests that, while important, such within-trial clustering effects are rarely taken into account in trials or in meta-analyses of these trials.

This paper proposes summary measures and individual-patient-data (IPD) methods for meta-analysing randomized trials with nested clustering effects. It extends methods for incorporating trials with unequal variances and cluster-randomisation to allow for between-trial and between-arm heterogeneity in ICC estimates. The work is motivated by a meta-analysis of trials of counselling in primary care, where the outcome of interest is the Beck Depression Inventory and the control is no counselling. We begin by outlining the example and presenting the statistical model at the trial-level. We then extend standard summary measures and IPD approaches to meta-analysis of mean differences and illustrate the proposed methods with our example. Fixed and random effects meta-analysis models are contrasted.

